# Frontostriatal grey matter atrophy in amyotrophic lateral sclerosis A visual rating study

**DOI:** 10.1590/1980-57642018dn12-040008

**Published:** 2018

**Authors:** Ratko Radakovic, Vaisakh Puthusseryppady, Emma Flanagan, Matthew C. Kiernan, Eneida Mioshi, Michael Hornberger

**Affiliations:** 1 Faculty of Medicine and Health Sciences, University of East Anglia, Norwich, UK; 2 Alzheimer Scotland Dementia Research Centre, University of Edinburgh, Edinburgh, UK; 3 Brain & Mind Centre, University of Sydney, Sydney, Australia; 4 Euan MacDonald Centre for Motor Neurone Disease Research, University of Edinburgh, Edinburgh, UK.

**Keywords:** amyotrophic lateral sclerosis, magnetic resonance imaging, orbitofrontal cortex, striatum, visual atrophy rating scale, esclerose lateral amiotrófica, ressonância magnética, córtex orbitofrontal, estriado, escala visual de atrofia

## Abstract

Amyotrophic lateral sclerosis (ALS) is characterised by frontostriatal grey matter changes similar to those in frontotemporal dementia (FTD). However, these changes are usually detected at a group level, and simple visual magnetic resonance imaging (MRI) cortical atrophy scales may further elucidate frontostriatal changes in ALS.

**Objective:**

To investigate whether frontostriatal changes are detectable using simple visual MRI atrophy rating scales applied at an individual patient level in ALS.

**Methods:**

21 ALS patients and 17 controls were recruited and underwent an MRI scan. Prefrontal cortex sub-regions of the medial orbitofrontal cortex (MOFC), lateral orbitofrontal cortex (LOFC) and anterior cingulate cortex (ACC), striatal sub-regions of the caudate nucleus (CN) and nucleus accumbens (NAcc) were rated using visual grey matter atrophy 5-point Likert scales.

**Results:**

Significantly higher atrophy ratings in the bilateral MOFC only in ALS patients versus controls was observed (p<.05). Patients with greater MOFC atrophy had significantly higher atrophy of the CN (p<.05) and LOFC (p<.05).

**Conclusion:**

Use of simple visual atrophy rating scales on an individual level reliably detects frontostriatal deficits specific to ALS, showing MOFC atrophy differences with associated CN and LOFC atrophy. This is an applicable method that could be used to support clinical diagnosis and management.

## RESUMO

A esclerose lateral amiotrófica (ELA) é caracterizada por alterações na substância cinzenta frontostriatal, semelhantes às da demência frontotemporal (DFT). No entanto, essas alterações geralmente são detectadas em nível de grupo, e as escalas simples de atrofia cortical por ressonância magnética visual (MRI) podem elucidar ainda mais as alterações frontostriatais na ELA.

**Objetivo:**

Investigar se as alterações frontostriatais são detectáveis usando escalas de classificação de atrofia MRI visuais simples aplicadas em um nível de paciente individual em ELA.

**Métodos:**

21 pacientes com ELA e 17 controles foram recrutados e submetidos a uma ressonância magnética. Sub-regiões do córtex pré-frontal do córtex orbitofrontal medial (MOFC), córtex orbitofrontal lateral (LOFC) e córtex cingulado anterior (ACC), sub-regiões estriadas do núcleo caudado (NC) e nucleus accumbens (NAcc) foram classificadas usando escalas de atrofia de substância cinzenta visuais de Likert de 5 pontos.

**Resultados:**

Observações de atrofia significativamente maiores no MOFC bilateral em pacientes com ELA versus controles foram observadas apenas (p <0,05). Pacientes com maior atrofia do MOFC tiveram atrofia significativamente maior do CN (p <0,05) e LOFC (p <0,05).

**Conclusão:**

O uso de escalas de avaliação de atrofia visuais simples em um nível individual detecta de forma confiável déficits frontostriatais específicos para ELA, mostrando diferenças de atrofia MOFC com atrofia associada de CN e LOFC. Este é um método aplicável que pode ser usado para apoiar o diagnóstico e o gerenciamento clínico.

Subcortical atrophy still remains largely clinically undetected in amyotrophic lateral sclerosis (ALS), despite recent evidence showing significant atrophy in subcortical regions.^1^^,^ 2 Subcortical changes should not be surprising in ALS, as frontotemporal dementia (FTD), which lies on a disease spectrum with ALS,^3^^,^ 4 also exhibits significant subcortical, and particularly frontostriatal atrophy in the early stages.^5^ The medial prefrontal cortex regions are strongly connected to striatal regions and previous research has shown that both have been associated with cognitive functioning and behaviour, which are often affected in FTD and ALS.^6^

However, detection of this frontostriatal atrophy clinically remains limited, as such changes have only been investigated at a group level^7^ and with imaging techniques that are difficult to be utilised clinically (e.g. voxel-based morphometry, cortical thickness). By contrast, visual magnetic resonance imaging (MRI) atrophy rating scales^8^ are reliable tools used in clinical practice for detection of atrophy at an individual patient level. Previous research using MRI rating scales has shown greater atrophy in the medial prefrontal cortex and orbitofrontal cortex in behavioural variant FTD (bvFTD) patients compared to Alzheimer’s Disease patients and controls.^9^^,^ 10 Further, in terms of the ALS-FTD spectrum, visual atrophy ratings showed that patients with ALS-FTD and bvFTD had higher orbitofrontal cortex, anterior cingulate cortex, motor cortex and anterior temporal lobe atrophy compared to controls.^10^ Notably, in this same study, patients with ALS only significantly differed to controls for the orbitofrontal cortex.

Therefore, we selected areas of the prefrontal cortex driven by previous research on the ALS-FTD spectrum,^9^^,^ 11 specifically the orbitofrontal cortex (medial and lateral subdivisions) and the anterior cingulate cortex. Further striatal areas were chosen based on previous research,^1^^,^ 5 specifically the caudate nucleus and nucleus accumbens. The current study aimed to investigate whether a clinically feasible visual MRI atrophy rating scale could be employed to reliably detect frontostriatal changes in ALS.

## METHODS

### Case selection

A total of 21 ALS patients without dementia were recruited from the Sydney ALS clinic, all fulfilling diagnostic criteria for ALS^12^ and ALS-FTD.^3^ Seventeen controls were also recruited from a healthy control panel. All participants underwent an MRI scan. Further, patients were rated on the ALS-Functional Rating Scale-Revised^13^ and Addenbrooke’s Cognitive Examination-Revised (ACE-R),^14^ and Cambridge Behavioural Inventory-Revised (CBI-R)^15^ scores were available. CBI-R scores were converted to percentages, as in previous research.^16^

Ethics approval for the study was obtained from the Human Research Ethics Committee of the South Eastern Sydney/Illawarra Area Health Service (HREC10/126). Consent was obtained from all participants following the ethos of the Declaration of Helsinki.

### Image acquisition and scan rating

All patients and controls underwent the same imaging protocol with whole-brain T1-weighted images using a 3-Tesla Philips MRI scanner with standard quadrature head coil (coronal orientation, matrix 256×256, 200 slices, 1×1 mm^2^ in-plane resolution, slice thickness 1 mm, TE/TR=2.6/5.8 ms, flip angle a=19°).

Two trained raters (RR and VP), blind to diagnosis, rated axial and coronal T1 MRIs on a 5-point Likert scale, with 0 indicating normal and 4 indicating severe atrophy using methodology described previously.^8^^,^ 11 We selected the medial orbitofrontal cortex (MOFC), lateral orbitofrontal cortex (LOFC) and the anterior cingulate cortex (ACC) as prefrontal cortex regions of interest. The boundary between MOFC and LOFC was differentiated using the crown of the gyrus rectus.^17^ In terms of the striatum, we selected the caudate nucleus (CN) and the nucleus accumbens (NAcc), which was identified using the rostrum of the corpus callosum as a marker. Based on prefrontal cortex and CN rating methods, we developed a visual rating for the NAcc (See Supplementary Figure 1 – available on the site www.demneuropsy.com.br/). Left and right regions were rated independently. For all of the scales, both raters showed excellent intra-rater reliability (RR Cronbach Standardised Alpha=0.93; VP Cronbach Alpha=0.92) and very good inter-rater reliability (Kappa=0.81, *p*<.001) on an independent MRI atrophy rating training set of 38 scans.

### Statistical analysis

Data was analysed using R statistical software. Shapiro-Wilk tests were used to examine normality of data. Parametric statistics (t test) were used to examine demographic variables, while the Chi squared test was used to compare gender distribution between patients and controls. Total brain atrophy was derived by adding the atrophy ratings of the MOFC, LOFC, ACC, CN and NAcc. Total prefrontal atrophy was derived by adding the atrophy ratings of the MOFC, LOFC and ACC, whereas total striatal atrophy was derived by adding atrophy of the CN and NAcc. Within-region visual atrophy rating differences and laterality were examined using Bonferroni-corrected Mann-Whitney U and the left-right average was taken for further comparison based on previous research.^11^ Spearman’s Rho was used for correlational comparisons.

## RESULTS

### Demographic and background comparison

There was no significant age or education difference between ALS patients and controls (Table 1). Patient and control groups differed in gender distribution.

**Table 1:** Demographics of ALS patients and controls.

	ALS (N=21)	Controls (N=17)	Test statistic	*p* value
Age (Mean, SD)	61.8 (13.1)	54.5 (14.3)	t=1.65	NS
Years of Education (Mean, (SD)	12.7 (3.6)	13.4 (1.8)†	t=0.26	NS
Gender (M/F)	10/11	2/15	χ^2^=5.59	< .05
Age at onset (Mean, SD)	59.0 (12.9)			
Disease duration, years (Mean, SD)	2.2 (2.1)			
Site of Onset (Bulbar/Limb)	7/14			
ALSFRS-R Total (Mean, SD) /48	38.7 (7.4)			
Bulbar subscore (Mean, SD) /12	8.6 (3.0)			
Fine motor subscore (Mean, SD) /12	9.0 (3.0)			
Gross motor subscore (Mean, SD) /12	9.7 (3.2)			
Respiratory subscore (Mean, SD) /12	11.4 (0.8)			
ACE-R (Mean, SD)/100	90 (9.0)			
CBI-R (Mean, SD) % deficit	14.6 (11.4)			

† N: 9; SD: Standard Deviation; ALSFRS-R: Amyotrophic Lateral Sclerosis Functional Rating Scale-Revised; ACE-R: Addenbrooke's Cognitive Examination-Revised; CBI-R: Cambridge Behaviour Inventory-Revised. Note: Age and Years of Education were normally distributed according to Shapiro Wilk tests (p > .05)

### Scan ratings

On this set of MRI scans, for all scales both raters showed excellent intra-rater reliability (RR Cronbach Standardised Alpha=0.95; VP Cronbach Alpha=0.94) and very good inter-rater reliability (Kappa=0.81, *p<*.001). Within-patient group gender comparison and within-control group gender comparison showed no significant difference between males and females in grey matter atrophy for the prefrontal cortex and striatum.

Overall, there was no significant difference in total brain atrophy, total prefrontal cortex and total striatum atrophy ratings, between patients and controls. On lateralization analysis, there were no significant differences in atrophy between left and right MOFC, LOFC, ACC, CN or NAcc. Subdivision of the prefrontal cortex showed significantly higher atrophy rating in MOFC in ALS patients versus controls (see Table 2). No significant atrophy differences were observed in LOFC, ACC, CN or NAcc between ALS patients and controls. No correlations were found between atrophy and the ACE-R and CBI-R.

**Table 2:** Scan Rating comparison (mean and standard deviation) between ALS and control groups

	ALS	Controls	Test statistic	*p* value
ACC	1.5 (0.7)	0.9 (0.6)	U=236.0	NS
LOFC	1.2 (0.7)	0.9 (0.7)	U=221.0	NS
MOFC	1.1 (0.6)	0.5 (0.5)	U=263.0	**< .05**
CN	1.4 (1.1)	0.8 (0.8)	U=217.5	NS
NAcc	1.6 (1.1)	0.7 (0.8)	U=188.5	

NS ACC: Anterior Cingulate Cortex; LOFC: Lateral Orbitofrontal Cortex; MOFC: Medial Orbitofrontal Cortex; CN: Caudate Nucleus; NAcc: Nucleus Accumbens; NS: Not Significant; Significant difference highlighted in bold. Mean (Standard Deviation) are shown. Note: Data were not normally distributed according to Shapiro Wilk tests (p<.05).

**Figure 1:**
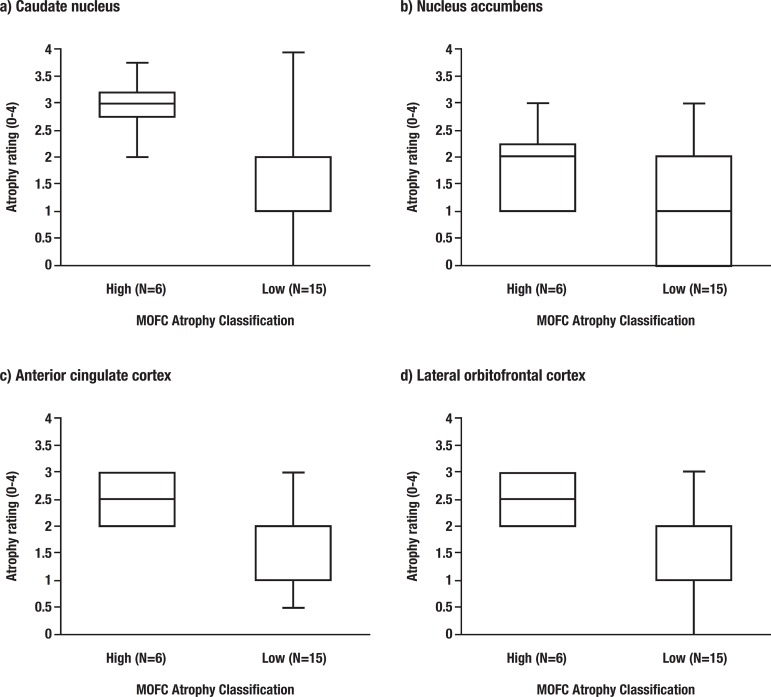
ALS patients with high and low medial orbitofrontal cortex (MOFC) atrophy comparison on striatal atrophy (caudate nucleus and nucleus accumbens) and prefrontal cortex atrophy (anterior cingulate cortex and lateral orbitofrontal cortex)

A further exploratory analysis of the differences based on MOFC atrophy and relating to other prefrontal and striatal areas was conducted. ALS patients were classified based on high and low MOFC atrophy using median split to explore striatal differences (Median=1), where patients with scores of<1 were classified as low MOFC atrophy and patients with scores of >1 were classified as high MOFC atrophy. ALS patients with higher MOFC atrophy tended to have significantly higher CN atrophy (*p<*.05) and LOFC atrophy (*p<*.05) than those patients with less MOFC atrophy, with no such differences observed in NAcc and ACC (see Figure 1). There was no significant difference between low MOFC and high MOFC atrophy on the ACE-R and the CBI-R.

## DISCUSSION

Our findings show significant atrophy to the medial portion of the OFC in ALS using visual atrophy rating methods applied at an individual case level. This builds upon previous research that showed the OFC was the only area where ALS patients had higher atrophy,^11^ and reoccurrence of atrophy in this area in FTD when compared to AD.^9^^,^ 10 Furthermore, this area has been shown to have greater grey matter atrophy in patients with FTD and ALS-FTD.^18^ Our findings point to pathological changes in ALS associated with specific regions of the OFC, which likely map onto the ALS-FTD subtypes.^3^^,^ 4

Additionally, ALS patients subdivided by MOFC atrophy provided partial insight into prefrontal and striatal changes, specific to the CN and LOFC. There is strong striatal structural and functional connectivity in this region,^19^ with observable associated white matter changes in ALS.^20^ Relevantly, in FTD, frontostriatal atrophy profile has been observed, including the MOFC, CN and NAcc regions, distinguishing these patients from controls and also Alzheimer’s disease patients.^5^^,^ 21 In terms of ALS, atrophy in the CN and OFC has been previously found,^1^^,^ 22 particularly in later stages of the disease.^23^ Additionally, reduced white matter integrity has been observed in ALS.^24^ Also, these frontostriatal regions have been found to be affected in ALS-FTD patients, with further impact on structural connectivity.^25^ Future research should aim to apply white matter visual atrophy rating scales (e.g.^26^), in parallel with grey matter atrophy rating, to further understanding of cortical and subcortical changes in ALS. As such, visually rated grey matter atrophy could be combined with visually rated white matter atrophy, with the possibility of composing a cumulative atrophy index that can be applied to connectivity between regions.

Regarding the study limitations, the sample size of this study was small and therefore replication of this methodology in studies with a larger sample size would be of importance, so as to further determine the visually rated cortical atrophy in ALS. Further, detailed phenotyping (i.e. genetic status and pathology) was not available and additional examination in a larger sample with mapping of ALS-FTD variants using validated tools (e.g. Edinburgh Cognitive and Behavioural ALS Screen^27^) onto the gradient of atrophy observed would further validate the visual rating and its clinical applicability. Our findings do, however, emphasise the sensitivity of visual atrophy rating to cortical and subcortical changes, which can be widely used in clinical practice.

In summary, our findings reinforce the clinical value and research impact of visual atrophy rating of MRI scans, with observable frontostriatal changes (notably the MOFC and related CN and LOFC) detectable in ALS. Further research should apply this visual rating method to explore connectivity between cortical and subcortical regions, accounting for white matter changes, to allow comprehensive visual atrophy rating. In both clinical and research settings, this is an accessible method that can help further our understanding of neuroanatomical changes on the ALS-FTD spectrum, whilst supporting diagnosis and management.
